# Evaluating a large language model-powered system for semi-structured interview in health research

**DOI:** 10.1093/jamiaopen/ooag218

**Published:** 2026-11-12

**Authors:** Janet Currie, Meirong Liu, Jae Eun Chung, Lu He, Jiang Li, Aaryan Panthi, Rahual Rai, Bibesh Sedhain, Chloe Riche, Olivia Kasule

**Affiliations:** Department of Economics, Yale University, New Haven, CT 06511, United States; Princeton School of Public and International Affairs, Princeton University, Princeton, NJ 08544, United States; School of Social Work, Howard University, Washington, DC 20059, United States; The Cathy Hughes School of Communications, Howard University, Washington, DC 20009, United States; School of Technology for Public Health, Arizona State University, Phoenix, AZ 85004, United States; Department of Electrical Engineering and Computer Science, Howard University, Washington, DC 20059, United States; Department of Electrical Engineering and Computer Science, Howard University, Washington, DC 20059, United States; Department of Computer Science and Engineering, University of California San Diego, La Jolla, CA 92093, United States; Department of Electrical Engineering and Computer Science, Howard University, Washington, DC 20059, United States; Department of Neuroscience, Princeton University, Princeton, NJ 08544, United States; Department of Neuroscience, Princeton University, Princeton, NJ 08544, United States

**Keywords:** artificial intelligence, large language model, semi-structured interview, health equity, community-based participatory research

## Abstract

**Objectives:**

Conducting qualitative health research with marginalized populations presents persistent challenges related to trust, disclosure, and resource constraints. Traditional in-person interviews can be costly and difficult to scale, while participants may hesitate to share sensitive experiences openly. This study assessed the feasibility and acceptability of a large language model (LLM)-powered conversational interview system as an alternative to semi-structured qualitative interviews with marginalized populations in a community-engaged health research context.

**Materials and Methods:**

We developed a web-based, LLM-powered interview system informed by a community-based participatory research framework. The system was tested with 28 caregivers of children with asthma living in subsidized housing. Postinterview evaluation sessions and researcher observation notes were analyzed to assess the system’s feasibility and acceptability and identify usability, accessibility, technical, and implementation considerations.

**Results:**

Participants reported that the system was easy to use and appreciated its empathetic and nonjudgmental tone. They also rated the system positively for clarity, comfort, and accessibility, indicating that conversational artificial intelligence (AI) systems can be adapted to support qualitative data collection in underserved communities. Identified challenges included intermittent technical errors, limited contextual adaptability, and variations in participants’ comfort with digital technology.

**Discussion:**

Large language model-powered conversational interview systems, when guided by community engagement, ethical design principles, and human support, offer a feasible and acceptable approach to qualitative health research with underserved populations. Hybrid human–AI models may offer a scalable yet inclusive pathway for expanding participation and advancing health equity in qualitative research.

**Conclusion:**

These findings support further development and evaluation of LLM-powered interview systems for community-engaged qualitative research. Ongoing technical improvement, ethical safeguards, and participatory design will be critical to ensuring these systems are used responsibly and effectively to advance health equity.

## Introduction

Recent advances in artificial intelligence (AI) are creating new opportunities for improving how researchers elicit perspectives and experiences. Large language models (LLMs) can simulate human-like conversations, making it possible to conduct in-depth interviews at scale without the high costs and logistical challenges of traditional methods.^1^ By enabling richer interactions and capturing respondents’ thoughts in their own words, LLMs have the potential to combine the depth of qualitative methods with the efficiency of large-scale data collection.^2^ This capability is particularly valuable in public health research, where understanding lived experiences is essential for designing effective interventions and policies.

Traditional methods involve a well-documented tradeoff between depth and scale. Structured surveys allow researchers to collect data from large populations, but they often fail to capture the nuances of respondents’ experiences. In contrast, unstructured or semi-structured interviews provide rich, in-depth insights but are resource-intensive and difficult to scale.^3^ As a result, researchers frequently face a choice between conducting small-scale, personalized interviews and collecting large-scale, standardized data.^3^ This tension highlights a clear need for innovative approaches that can preserve the richness of qualitative insights while enabling broader reach—an important consideration in public health research.

Artificial intelligence-driven methods, particularly those using LLMs, may offer a solution to this challenge.^3–6^ Wuttke et al^3^ found that AI conversational interviewing could produce data comparable in quality to traditional interviews, while also offering greater scalability. Similarly, Chopra and Haaland^4^ used an LLM to conduct qualitative interviews and reported that it produced rich, high-quality data at a fraction of the cost of human-led interviews. Fieldhouse et al^5^ highlighted the growing use of AI to support qualitative inquiry and improve methodological efficiency. At the same time, a large-scale evaluation of LLM-based interviewing chatbots found that while these systems performed well on established quality metrics, they often failed to capture contextual richness, personalized motives, and nuanced social meaning, underscoring important limitations in scaling qualitative research with LLMs.^6^ Despite these advances, the feasibility and acceptability of LLM-powered conversational interviews specifically within underserved communities remain largely unexamined, representing an important gap for equitable and inclusive data collection in public health research.

The present study addresses this gap through an exploratory pilot examining the feasibility and acceptability of an LLM-powered interview system to support scalable, resource-efficient qualitative data collection while maintaining the depth and quality of insights essential to public health research. We developed a culturally responsive LLM-powered interview system and evaluated its use with caregivers of children with asthma living in subsidized housing—an underserved community whose experiences are often underrepresented in health research. The focus on caregivers of children with asthma in subsidized housing was grounded in prior community-based participatory research (CBPR) work in Washington, DC,^7^ which highlighted the disproportionate burden of asthma in low-income housing and caregivers’ central role in its management. This work also revealed persistent barriers to engaging this population in traditional qualitative interviews, including time constraints and limited flexibility. These insights motivated our focus on this underrepresented group and informed our exploration of whether an LLM-powered interview system could offer a more scalable, lower-burden approach to capturing caregivers’ lived experiences.

The objectives were to: (1) assess the feasibility and acceptability of using an LLM-powered semi-structured interview system and (2) identify usability, accessibility, technical, and implementation considerations for using LLM-powered interviews with marginalized populations in public health research. Rather than introducing a novel interview architecture, this study examines whether LLM-powered conversational systems can be effectively adapted for qualitative health research with underserved populations. Specifically, we focus on caregivers of children with asthma living in subsidized housing and evaluate how community-informed design and implementation strategies influence feasibility, acceptability, accessibility, and participant engagement. A particular emphasis of this study is understanding participants’ experiences with the AI interviewer itself, including perceptions of comfort, trust, responsiveness, accessibility, and preferences regarding AI-mediated vs human-led interviewing.

## Literature review

### Constraints of qualitative research with hard-to-reach populations

Qualitative methods help contextualize health-related experiences, including social determinants, health-care-seeking behaviors, and barriers shaping interventions.^5^ However, conducting traditional in-depth interviews with marginalized populations—such as caregivers of children with asthma in subsidized housing—poses challenges. These caregivers often face time constraints from caregiving and employment demands, economic pressures, and limited flexibility for scheduled research, while distrust of researchers, concerns about confidentiality and potential repercussions, and the sensitivity of discussing stigmatized or resource-related issues further hinder participation.^8–10^ As a result, this population—despite bearing a disproportionate burden of pediatric asthma—remains underrepresented in research.

At the same time, asthma management in subsidized housing is closely tied to environmental conditions, requiring nuanced, experience-based accounts. While traditional interviews yield rich data, they are time-intensive, geographically constrained, and difficult to scale, limiting broader inclusion. These challenges motivated us to explore LLM-powered interview systems as a flexible, lower-burden approach to expand reach while preserving depth.

### Promises of LLM-powered interviews in health research

Large language model-powered tools can support scalable qualitative inquiry by enabling analysis of unstructured narrative data while preserving contextual sensitivity.^5^^,^^11^ They may expand engagement with marginalized communities by reducing participation barriers and capturing diverse experiences that are often excluded from conventional approaches.^8^^,^^11^

By allowing flexible, self-paced participation, these systems reduce logistical constraints such as scheduling and transportation,^12^ while improving consistency in question delivery and follow-up, potentially minimizing interviewer bias.^3^^,^^4^ They can also adapt language and tone to participants’ cultural contexts, supporting more inclusive and empathetic communication.^13^ When designed with appropriate safeguards and community input, such tools can expand research reach, facilitate engagement with underrepresented populations, and complement traditional human-led methods through scalable, efficient, and participant-centered approaches.^13^

For individuals discussing sensitive or stigmatized issues, LLM-powered systems can offer a less intimidating and more private environment, helping reduce perceived power imbalances and enabling more open expression without fear of judgment or stigma.^14^ The anonymity and privacy offered by AI-mediated communication may foster greater openness when discussing topics related to health, poverty, or social marginalization.^15^ Large language model-powered systems can also be adapted for different literacy levels and modes of expression—such as voice or text. Ultimately, LLM-powered interview systems can support equitable participation and amplify the voices of those who are often underrepresented in health research.^10^

Recent work has begun to apply LLM-powered conversational systems in health-related contexts. Jo et al^16^ deployed an LLM chatbot for public health monitoring of socially isolated individuals; Sezgin et al^17^ designed a chatbot for social needs screening with vulnerable families; Li et al^18^ evaluated LLM-powered chatbots for structured information gathering in clinical preconsultation; and Liu et al^19^ developed humanized LLM conversational agents for hospital admission interviews. While these studies demonstrate the promise of LLM-driven systems across diverse health service contexts, they primarily focus on structured tasks such as screening or intake. In contrast, the present study examines their use as a semi-structured qualitative interviewing tool within a community-based participatory framework—an application that emphasizes open-ended narratives, community engagement, and health equity. This represents the primary gap addressed in this study. Existing studies have largely focused on structured screening, intake, monitoring, or information-gathering tasks, whereas little is known about how LLM-powered interviewing can be adapted for community-engaged qualitative research involving marginalized populations. Furthermore, prior work provides limited guidance regarding the accessibility, cultural responsiveness, and implementation supports needed to deploy such systems among caregivers facing significant socioeconomic and health-related challenges. In addition, relatively little research has examined how participants themselves perceive and experience LLM-powered interviewers, particularly within underserved communities where trust, accessibility, and comfort may influence willingness to participate in research.

### Barriers to AI adoption for marginalized communities

Pronounced differences in digital literacy, technology use, and attitudes toward AI persist across social and economic groups. Lower-income, older, and less-educated individuals tend to have lower familiarity, limited digital access, and skill levels,^20^ which contribute to lower digital confidence.^21^ Digital confidence also moderates the relationship between people’s experiences with everyday AI technologies and their overall attitudes toward AI.^21^ Negative digital experiences further diminish trust and willingness to adopt new technologies perpetuating a cycle of exclusion.^21^ Even when devices and internet access are available, actual use of digital tools for important tasks (eg, health information) is low in lower-income groups.^22^ Additionally, AI systems are frequently developed without meaningful input from the marginalized communities they are intended to serve, resulting in tools that may overlook real needs or unintentionally reproduce harm.^23^ These dynamics compound broader inequities, as marginalized groups have been underserved by emerging technologies,^24^ and early gaps in technology adoption can worsen health disparities.^25^^,^^26^

### Core ethical principles and community-engagement AI for health research

Previous scholars have identified 4 core ethical principles for the development and deployment of AI in health research: (1) justice (promoting fairness and equitable access), (2) beneficence (prioritizing human well-being and social benefit while preventing harm), (3) autonomy (protecting individual decision-making), and (4) explicability (ensuring transparency and accountability).^27^^,^^28^ Recent work further emphasizes the importance of data quality, transparency, and strong ethical governance throughout the design and deployment process.^29^^,^^30^ Central to these efforts is meaningful community engagement. Incorporating CBPR principles can help mitigate risks of bias and inequity while enhancing cultural relevance, trustworthiness, and accessibility of AI-driven approaches.^31^

### The central motivation for the present study

Despite the promise of AI-enabled tools, significant concerns persist regarding accessibility, trust, empathy, and sustained engagement—especially among populations historically marginalized in research. Uncertainty also remains about whether LLM-powered interview systems can generate prompts and responses that are contextually appropriate, culturally sensitive, and capable of amplifying the voices and experiences of underserved communities.

## Methods

### Development of the LLM-powered interview system

#### Community-engaged design

This project builds on a prior interdisciplinary CBPR initiative, led by a team across a Historically Black College or University and a Predominantly White Institution. The broader initiative focused on improving the health of asthmatic children living in subsidized housing. Across prior activities—including surveys and semi-structured interviews—community engagement was central. This approach guided the design and development of the LLM-powered interview system. The semi-structured interview protocol (Appendix S1), originally developed for in-person interviews and reused in this study, focused on caregivers’ lived experiences—including access to Healthy Home services, perceptions of program implementation, and resource needs for equitable asthma care.^7^ Interview questions were refined through iterative feedback from community partners to ensure content relevance, cultural and linguistic appropriateness, and were piloted with participants from prior human-led interviews. The research team also maintained ongoing engagement with community partners by participating in community events (eg, cultural gatherings, health affairs, local and national resident council meetings), supporting reciprocal relationships and positioning community members as partners.^31^ These interactions provided first-hand insights into participants’ environments, technology preferences, and comfort with digital tools, including interest in AI for health communication.

#### Iterative design process: prototype testing and refinement

The LLM-powered interview system, *Asthma Care Talk*, is a web-based interactive system designed to engage participants in semi-structured interviews. Its prompt design incorporated deidentified summaries from prior human-led interviews to reflect participants’ conversational patterns and language choices (eg, short sentences, simple vocabulary, and a culturally appropriate, respectful tone).

Google Gemini 2.0 Flash^32^ was used as the LLM backbone for the final deployed system. During development, the research team reviewed alternative LLM options, including OpenAI o3-mini.^33^ The final selection was guided by practical implementation considerations, including response latency, readability, conversational flow, and deployment feasibility.

Initial prototypes were tested by researchers and community partners to assess usability, clarity, tone, and accessibility. Feedback informed successive rounds of refinement, including adjustments to prompt wording, question flow, interface layout, and accessibility features. Early feedback identified issues with readability, navigation, and text input burden, leading to improvements such as larger fonts, higher contrast, culturally familiar visual elements, and speech-to-text functionality. Throughout the iterative design process, prompts were systematically tested and refined to ensure the system generated empathetic, engaging, and responsive responses while adhering to the intended semi-structured interview protocol.

The system used a 3-layer prompt architecture: behavioral, validation, and content. The behavioral layer instructed the model to act as a friendly, culturally responsive social science interviewer, asking 1 question at a time, using plain language, and maintaining a supportive, nonjudgmental tone. The validation layer guided the system in assessing whether participants had responded to the prior question and remained within the scope of the interview. Specifically, the model was instructed to evaluate whether a response captured the core intent of the interview question and broadly aligned with the interview guide. Responses did not need to be complete or highly detailed, but they were required to be relevant to the topic. If a response was judged to be relevant, the system advanced to the next question. If a response was unclear or off-topic, the system generated a brief clarification or redirection prompt before continuing. Prompt-level safeguards also instructed the model to decline requests for clinical or medical advice and direct participants to qualified health-care providers instead. The content layer incorporated a master interview guide covering Healthy Home programs, while drawing on scenarios from prior human-led interviews to reflect caregivers’ lived experiences and communication needs. This iterative cycle supported the development of an interview system that remained accessible, culturally responsive, and aligned with participant needs. The prompt integrating content from the 3 layers is provided in a public GitHub Repository (see “Data Availability”).

### LLM-powered semi-structured interviews

#### Participants

Participants were caregivers of children with asthma living in subsidized housing in Washington, DC, recruited through community partnerships. A total of 29 caregivers participated (July-August, 2025). All were newly recruited and had not previously participated in the earlier human-led interviews, avoiding repeated-testing bias and carryover effects.

#### Procedure

Interviews began with a system demonstration and informed consent. Participants could skip questions or withdraw at any time. The semi-structured interview protocol is provided in Appendix S1. During sessions, 1 researcher provided technical support, and another conducted structured observations, documenting usability issues, participant comfort, and engagement using a researcher observation form adapted from Wuttke et al^3^ (Appendix S2). Because the present manuscript focuses on feasibility and acceptability, the findings reported here are based on postinterview evaluation data rather than thematic analysis of the interview conversation logs.

#### Postinterview evaluation

Participants completed a structured in-person evaluation informed by Wuttke et al,^3^ assessing question clarity, ease of use and navigation, comfort with AI-generated questions, repetition, whether participants felt heard, preferences for human vs AI interviewers, and suggestions for improvement. Closed-ended items used a 5-point Likert scale ranging from strongly disagree to strongly agree. The postinterview evaluation form is provided in Appendix S3. Evaluation sessions were audio-recorded for analysis. Following the interview, participants received asthma education materials, including videos narrated by a certified pediatric asthma educator and a flyer with a QR code linking to the full video library. The study procedure is illustrated in Figure 1 and was approved by the university’s Institutional Review Board.

**Figure 1.**
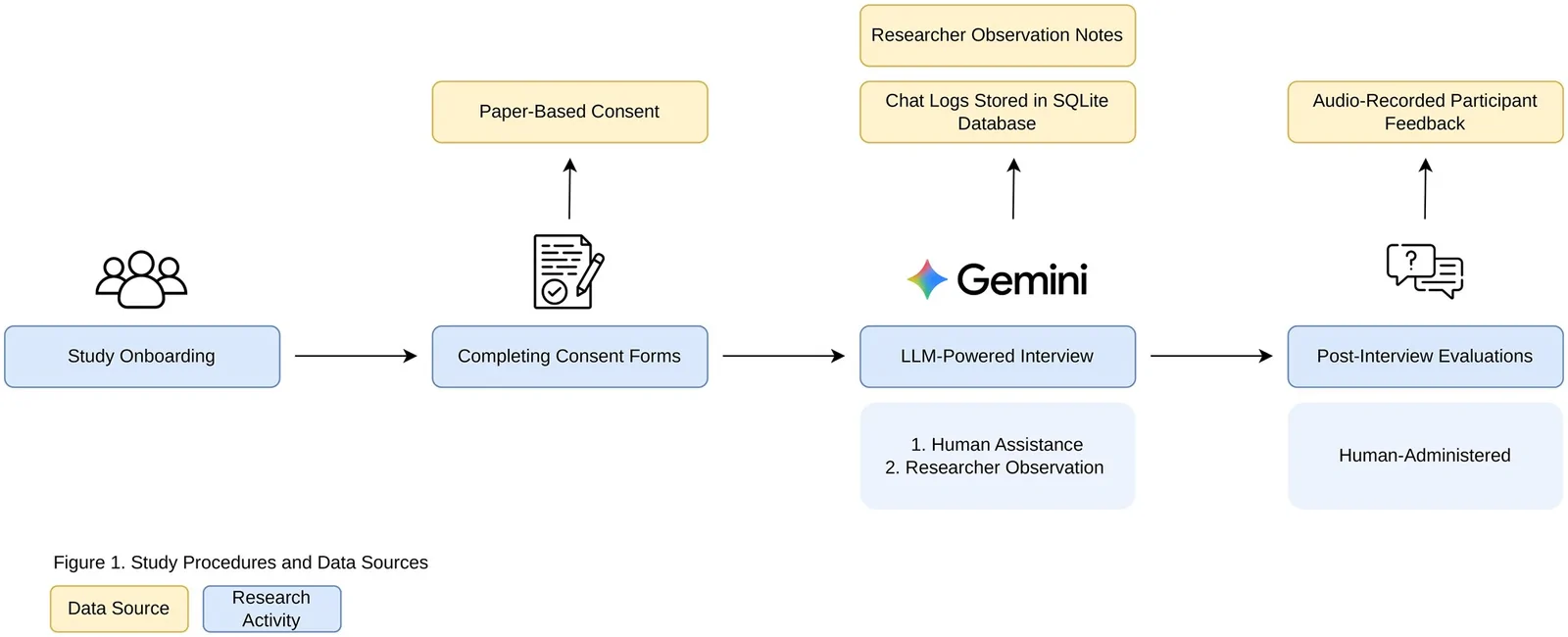
Study procedures and data sources.

### Data analysis

Analysis focused on postinterview evaluation data. Audio-recorded evaluations were transcribed using Otter.ai and verified for accuracy. Closed-ended responses were summarized with frequencies and percentages, with “agree” and “strongly agree” grouped as positive. Open-ended responses were analyzed thematically to contextualize ratings and identify themes related to feasibility and acceptability.

Thematic analysis followed established approaches by Jowsey et al^34^ and Nowell et al^35^ Three coders independently conducted open coding and developed preliminary code lists. Through iterative discussion, a consensus-based codebook was developed and applied using NVivo 14.0.^36^ Discrepancies were resolved through consensus meetings.

Open coding of the 29 evaluation transcripts generated 32 preliminary codes, which were refined into 13 codes across 6 themes. Findings were synthesized narratively by representative quotes.

## Results

### Participants’ characteristics

Most participants were female (72.4%). Ages ranged from the early 20s to over 60, with the largest groups aged 40-49 (27.6%) and 20-29 (24.1%) years. Most had a high school diploma or General Educational Development (GED) (62.1%), with others reporting some high school (20.7%) or some college (17.2%). Household incomes were low, with 41.4% earning $10 000 or less per year and 34.5% earning $10 001-$25 000. Public assistance use was common, with over half receiving the Supplemental Nutrition Assistance Program (SNAP) (51.7%) and others receiving Temporary Assistance for Needy Families (TANF), SNAP, or Supplemental Security Income (SSI). Most were single or never married (72.4%), and 41.4% were employed. Demographics are summarized in Table 1.

**Table 1.** Caregivers’ demographic characteristics (*n *= 29).

	*n*	%	*M*	SD
**Gender**				
** Female**	21	72.4		
** Male**	7	24.1		
** Missing/not reported**	1	3.4		
**Relationship to child(ren)**				
** Mother**	13	44.8		
** Father**	7	24.1		
** Grandparent**	8	27.6		
** Missing/not reported**	1	3.4		
**Age (year)**			44.5	15.2
** 20-29**	7	24.1		
** 30-39**	4	13.8		
** 40-49**	8	27.6		
** 50-59**	5	17.2		
** 60+**	5	17.2		
**Level of education**				
** Some high school**	6	20.7		
** High school diploma/GED**	18	62.1		
** Some college**	5	17.2		
**Household budget**				
** $0-10 000**	12	41.4		
** $10 001-$25 000**	10	34.5		
** $25 001-$40 000**	2	6.9		
** $40 001-$75 000**	1	3.4		
** Missing/not reported**	4	13.8		
**Public assistance**				
** SNAP**	15	51.7		
** TANF and SNAP**	7	24.1		
** SSI**	4	13.8		
**Marital status**				
** Single/never married**	21	72.4		
** Widowed/divorced**	6	20.7		
** Married**	1	3.4		
** Missing/not reported**	1	3.4		
**Employment status**				
** Employed**	12	41.4		
** Unemployed ≥1 year**	9	31.0		
** Unemployed <1 year**	2	6.9		
** Homemaker**	1	3.4		
** Other**	4	13.7		
** Missing/not reported**	1	3.4		

Sample size varies across categories due to missing or unreported responses. Percentages are calculated based on the full sample (*N* = 29).

### Caregivers’ experiences with the AI-powered interview process

Postinterview evaluation findings are organized around caregivers’ perceptions of clarity, comfort, accessibility, perceived responsiveness, technical challenges, and preferences for human vs AI interviews. A comprehensive summary table of thematic findings and representative quotes is provided in Appendix S4.

#### Clarity and comprehension of prompts

Nearly all participants (25, 86.2%) found the interview questions generated by the AI-powered system easy to understand. They described the questions as “simple and direct to the point” (CG03) and appreciated that “it wasn’t a bunch of big words that we wouldn’t understand” (CG25). Others emphasized that the system provided helpful follow-up prompts and clarifications: “It stayed on topic and followed up when I needed to explain something” (CG19).

#### Comfort and emotional engagement

Most participants (27, 93.1%) felt comfortable during the AI-powered interviews, describing the questions as noninvasive and relevant to their lived experience. One caregiver reflected, “It was easy to talk because it asked things I actually know about—my child and our home” (CG19).

Participants attributed their comfort to both the system’s empathetic tone and the support provided by researchers present during the sessions. As one caregiver said, “It felt natural, like I was just having a conversation—but someone was there if I got stuck” (CG14).

Several participants noted that the system “didn’t judge” (CG09) and that its neutral style made them feel more at ease. For example, 1 caregiver shared, “I felt like I could say what I needed to without worrying someone would think I wasn’t doing enough” (CG27).

#### Ease of navigation and accessibility

Caregivers consistently described the system as easy to use and intuitive. Participants cited features such as clickable buttons, visual layout, and voice options as key elements that enhance accessibility. One caregiver explained, “I liked that it read the questions out loud—I could just talk instead of typing” (CG07). Another participant, new to using computers, noted, “Even though I don’t use computers much, this one was simple enough for me to figure out” (CG29).

#### Perceived responsiveness and feeling heard

A central strength of the AI-powered interview was its perceived responsiveness. Most participants felt that the system “listened” and acknowledged their experiences. They described the interaction as “like talking to someone who really listens” (CG26) and “felt like a real conversation” (CG12).

Participants also reported that the system provided clarifications and follow-up questions at appropriate moments: “It repeated me and then piggybacked—it was a good thing, like it was paraphrasing what I said” (CG12).

However, some participants perceived occasional repetition as unnecessary: “It started kind of being repetitive, like it wasn’t hearing what it wanted to hear” (CG26).

#### Technical challenges and barriers

A few participants experienced challenges with literacy, vision, and computer familiarity. For instance, 1 caregiver shared, “I had trouble seeing the screen sometimes, but it was okay once I figured out the voice part” (CG03). Another noted, “I’m not too good with computers, but it was easy after a few tries” (CG15).

Minor technical pauses or delays were mentioned by some (CG14, CG28) but were generally described as manageable and did not detract from the overall experience. A small number of participants expressed skepticism about AI’s role in research, stating, “It’s not the same as a person who can really understand” (CG22).

#### Human vs AI preference

Responses were mixed regarding participants’ preference between human and AI interviewers. Nearly half (44.8%) preferred human interviews, citing a desire for personal connection—“I like talking to a real person because they can understand better” (CG07)—while one-third (34.5%) appreciated both formats, noting complementary strengths. As 1 caregiver put it, “AI doesn’t judge like people do, but people can help more” (CG09).

A smaller portion (17.2%) preferred the AI-led interview, describing it as comfortable, straightforward, and nonjudgmental. One participant reflected, “This is actually my first good AI experience—I’m old school, but maybe we can give it a chance now” (CG12).

## Discussion

### Feasibility and acceptability of LLM-powered interviews

This study provides preliminary, uncontrolled evidence that an LLM-powered interview system can be a feasible and acceptable way to engage caregivers of children with asthma in subsidized housing. The contribution of this work lies not in the development of a new LLM architecture, but in demonstrating how conversational AI interviewing can be implemented within a CBPR framework to support qualitative health research with an underserved population. Participants consistently rated the system positively for clarity, comfort, and accessibility, indicating that conversational AI systems can be adapted to support qualitative data collection in underserved communities. These findings extend prior work suggesting that empathy, simplicity, and transparency in AI design improve user engagement and trust.^37^^,^^38^

Importantly, while prior studies have examined LLM-powered conversational agents for public health intervention,^16^ social needs screening,^17^ and clinical information gathering,^18^^,^^19^ this study examines a distinct application: using an LLM-powered system as a semi-structured qualitative interviewing tool within a community-based participatory research framework. This approach is applied to caregivers of children with asthma in subsidized housing—an underserved population often underrepresented in health research.^37^ Caregivers perceived the system as easy to use, aligned with their everyday language use, and nonjudgmental, reinforcing previous findings on the importance of linguistic accessibility and conversational tone for engagement.^12^ Collectively, the findings suggest that LLM-powered interviews, when designed with readability and empathy in mind and deployed in community health contexts, can create an inclusive communication space even for participants with limited literacy or technical skills.

### Community-engaged and culturally responsive design

Community voices shaped interview content, linguistic framing, interface design, and usability and accessibility features, reflecting calls for ethical and participatory AI design.^13^^,^^30^ By integrating community knowledge throughout the design process rather than a post hoc adaptation, this study demonstrates how community engagement functions as both an ethical commitment and a practical design strategy for developing culturally grounded and trustworthy systems.

### Human support and hybrid model

The human–AI hybrid design used in this study integrates the strengths of both human support and machine consistency.^1^ Participants described gaining confidence through human assistance with navigation, technical issues, and reassurance during the interview process. These findings mirror evidence that low-income and older adults often require scaffolding to engage meaningfully with digital platforms.^38^^,^^39^ While LLMs can standardize data collection and reduce interviewer bias, human presence ensures relational trust, emotional support, and ethical oversight—factors essential for research in underserved communities.

### Implications

This study advances understanding of AI-mediated qualitative research by showing that LLMs, when guided by ethical principles and community engagement, can function as socially responsive interview tools. Prior work highlights the role of algorithmic empathy and linguistic framing in shaping participant openness.^40^ Our findings suggest that community-informed, ethically designed systems are perceived by participants as supporting authentic dialogue and demonstrate promising potential for qualitative data collection with underserved populations. Whether this translates into dialogue of comparable depth to human-led interviews remains an important question for future research.

Our study followed ethical principles of justice, beneficence, and autonomy.^27^^,^^28^ Justice was addressed by reducing exclusion through community participation. Beneficence was supported through culturally responsive prompts, human assistance, and education materials to inform equitable programs for children in subsidized housing. Autonomy was upheld through transparent consent and participant control over pacing (the ability to pause, skip, or withdraw). Together, these measures demonstrate responsible AI use to promote inclusion, respect, and equity in health research.

### Challenges and future directions

Despite strong feasibility and acceptability, challenges remained. Participants noted occasional repetition, pauses, and minor technical glitches, common in conversational AI.^41^ Some participants also expressed uncertainty about initial interactions with the AI, though comfort generally increased over time. These observations emphasize the need for iterative system refinement, particularly response timing, adaptive follow-up, and contextual sensitivity. Such refinement should continue to engage community partners as co-designers, consistent with CBPR principles of shared power and community-driven research.^42^

The speech-to-text functionality was widely appreciated, particularly by participants with lower literacy or vision challenges, supporting prior evidence that multimodal interaction enhances accessibility.^43^ Future development should continue to prioritize user-centered enhancements that enable the system to adapt dynamically to different communication preferences and cultural contexts. Because this study was designed to assess feasibility and acceptability rather than to compare interview modalities, future research should use matched or comparative designs to examine differences in depth, rapport, data quality, participant comfort, scalability, and cost-effectiveness across LLM-powered and human-led interviews.

### Limitation

First, it relied on a small convenience sample in a single community setting in Washington, DC, limiting generalizability to other populations and contexts. Second, participants were aware of the study context, and research team members were present, which may have introduced social desirability bias or observation effects; future studies could use anonymous or independent postinterview evaluations to reduce these biases. Third, because this study reports only postinterview evaluation data, all claims regarding data richness, depth, or detail refer to participants’ perceptions of the interview process rather than to an independent analysis of interview content; thematic analysis of the interview conversation logs was outside the scope of this paper. Fourth, this study did not include a parallel human-led interview arm; consequently, feasibility and acceptability findings should be interpreted as uncontrolled observations of the AI-led approach on its own terms, not as evidence of comparative advantage or equivalence relative to human-led interviews.

## Conclusion

In sum, this study provides preliminary, uncontrolled evidence that LLM-powered interview systems, when guided by community engagement and ethical AI principles, are perceived by caregivers in underserved communities as a feasible, acceptable, and inclusive method of engagement. The interview system demonstrated clarity, accessibility, and relational warmth, and the hybrid human–AI model supported participant comfort and trust. The findings underscore the potential of LLM-powered interviewing to broaden participation in health research and amplify voices that are often underrepresented. Continued technical refinement, ethical oversight, and participatory design will be essential for the responsible and effective use of these systems in advancing health equity.

## Supplementary Material

ooag218_Supplementary_Data

## Data Availability

The prompt used in the LLM system is publicly available through GitHub: https://github.com/lijprof/SurveyGeminiPublic.
