# Supplementary material for: Evaluating a large language model-powered system for semi-structured interview in health research
Source: JAMIA Open. 2026 Nov 12;9(6):ooag218. doi: 10.1093/jamiaopen/ooag218 (PMC13755875; doi:10.1093/jamiaopen/ooag218)
Supplement: Supplementary file 1 [file ooag218_supplementary_data.docx]

**Supplementary Materials**

**Appendix 1**: **Semi-Structured** **Interview Protocol**

This appendix presents the full semi-structured interview protocol used to guide conversations with participants.

Can you introduce yourself and provide a little background on yourself and your experience of taking care (being a caregiver) of child(ren) with asthma?

1. Questions on Healthy Home programs 
1) Have you heard about or known the Healthy Home programs/services to identify and reduce at- home environmental asthma triggers?

a) If yes, can you tell us more about the services you know, or your family received? (*Probing question: What services were provided and how were such services provided/delivered?)*
b) Have you found such services helpful? If so, how? (*Probing question: e.g., reduced indoor asthma triggers, provided remediation resources, etc*). If not, how?
c) Thoughts or opinions as to the ways the services are implemented or monitored (key)?
d) How do you think such services were delivered to or received by the residents?

2) What services and resources can housing agencies, property managers, landlords, health providers, and community-based organizations provide to effectively reduce at-home asthma triggers?

2. Questions on Asthma Action Plan

1)  Have you ever heard of an Asthma Action Plan (AAP)? Have you had or do you have one for your child?

2) If yes, has the action plan been helpful in asthma management for your child(ren)? If you have or had an AAP, how easy or difficult was it to adhere to the plan? What types of support or resource can help caregivers, like yourself, adhere to the AAP?

3)  What do you think of the process of establishing the Asthma Action Plan? If you do not have an action plan, do you see any needs? If so, what types of support or resources can be helpful in developing an AAP?

3. Questions on Culturally Sensitive Education to Improve Self-management Skills

1)  What do you want to learn more about regarding your child’s asthma? What educational resources /programs can help your family to better understand and manage your child’s asthma?

2)  What is the best channel to deliver such educational resources/programs?

3)  What are some barriers to using educational resources/programs, if you were given free access?

4. Housing agencies, property managers/landlords, health providers, and community-based organizations are encouraged to provide comprehensive, sustainable, and equitable asthma care programs. Do you have any suggestions on how such efforts?

**Appendix 2:** **LLM Interview Researcher Observation Form**

This appendix includes the structured observation form used by researchers during LLM-facilitated interviews*.*

During the interview, please document any technical issues, unexpected behaviors, or participant confusion you observe. Add comments as needed.

1. Technical Issues: Document any technical issues during the interview, for example, problems with audio recording, excessive latency of AI Interview (response times), etc.

2. Respondent Uncertainty or Difficulty: Document when and why the respondent is unsure about what is expected or how to proceed.

3. Unexpected or Undesired AI Interviewer Behavior: Document any odd, unexpected, or undesired interviewer behavior that is inconsistent with interview guidelines.

**Appendix 3:**  **LLM Post-Interview Evaluation Form**

This appendix contains the post-interview evaluation instrument completed by participants following the LLM-based interview.

Thank you for participating in this session. As you use the system, I’d like you to think out loud - share what you're trying to do, what you're thinking, and anything that seems confusing or unexpected.

1. Were the AI’s questions easy to understand? Why?

(1 = Strongly Disagree | 2 = Disagree | 3 = Neutral | 4 = Agree | 5 = Strongly Agree)

2. Was the system easy to use (interface, navigation, starting and ending the interview)? How so?

(1 = Strongly Disagree | 2 = Disagree | 3 = Neutral | 4 = Agree | 5 = Strongly Agree)

3. Were there moments when the AI repeated itself or seemed off-topic? Why?

(1 = Strongly Disagree | 2 = Disagree | 3 = Neutral | 4 = Agree | 5 = Strongly Agree)

4. Did you feel comfortable answering the AI’s questions? How so?

(1 = Strongly Disagree | 2 = Disagree | 3 = Neutral | 4 = Agree | 5 = Strongly Agree)

5. Did the interviewer make you feel like your experiences mattered? How so?

(1 = Strongly Disagree | 2 = Disagree | 3 = Neutral | 4 = Agree | 5 = Strongly Agree)

6. What did you like most about your experience?
7. Which do you prefer, human or AI, how so? (Or: Do you find this AI tool engaging/would you prefer to talk to a real person instead?)
8. What did you dislike most? Are there any improvements you would suggest?

**Appendix 4.** **Thematic Findings and Representative Quotes from Caregivers’ Experiences with the LLM Interview Process**

| **Evaluation Questions** | **Representative Quotes** |
| --- | --- |
| **Were the AI’s questions easy to understand?** | |
| Clarity and simplicity of language | “Everything was worded understandable. Wasn’t hard to understand the questions, so it made the questions easier to answer.” (CG 05)  “It wasn’t a bunch of big words that we wouldn’t understand.” (CG 25)  “They were easy, simple and direct to the point.” (CG 14) |
| Health-related comprehension barriers | “Most of it was [easy] but I have seizures, so it’s hard for me to comprehend. Sometimes it’s hard for me to even remember.” (CG 20) |
| **Was the system easy to use (interface, navigation, starting and ending the interview)?** | |
| Ease of navigation and visual layout | “It was straight and simple” (CG 14)  “It was easy to use. Everything was step by step.” (CG 16)  “Because the buttons and stuff were there for you to just go to the next and very easy.” (CG 06)  “You just type your answer, or you speak and listen.” (CG 28) |
| Voice function and accessibility | “It was easy to navigate because everything was voice control” (CG 09)  “It read it to me, so I understood it better.” (CG 06)  “Because it read the questions for you, so you can sit back and really understand.” (CG 09) |
| Learning curve and human assistance | “Easy, because you did a wonderful job teaching me.” (CG 11)  “I never knew how to navigate from one part to another… and when y’all came, I just figured it all out.” (CG 07) |
| Minor Technical/Device Issues | “Computer was pretty tough because I had to redo it. Might just be my computer. Neutral.” (CG 23) |
| **Did the interview make you feel like your experiences mattered?** | |
| Validation and affirmation | “Yes, like he was being there, concerned and all that. And I give that a five.” (CG 01)  “Yes, it made me feel like my experience matters, and they cared about my experiences.” (CG 09)  “It did by reiterating my answers and making me understand they knew what I was talking about.” (CG 25)  “Yes, because it kept validating everything that I was saying and providing me with reassurance.” (CG 26) |
| Personal responsiveness | “It made me feel like it was a person: ‘Oh, that was a good idea… did you know about this?’” (CG 12)  “Yes, because it was like responding to what I was saying about my daughter.” (CG 16) |
| Skepticism about AI empathy | “I don’t feel like AI cares. It’s there to do a job. If you [human] asked me, I’d feel you care. I know it doesn’t care.” (CG 22)  “It felt like I was talking to someone real for the first half. But then towards the end, it kind of felt like I was talking to a bot.” (CG 26) |
| **Did the AI repeat itself or go off-topic?** | |
| Stayed on topic | “It never went off topic at all.” (CG 01)  “No, it was spot on.” (CG 06)  “It never go off topic. All the questions were related to asthma.” (CG 27) |
| Repetition as validation or paraphrasing | “It was reiterating. It repeated me and then piggybacked. It was a good thing, paraphrasing what you were saying.” (CG 12)  “it seemed like I was talking to a regular person… like piggybacking.” (CG 12)  “It looked like it was repeating itself, but it was just asking me the same thing in another way.” (CG  19)  “It was asking me like the same question I wasn’t recognizing.” (CG 28) |
| Repetition-related frustration | “It started kind of being repetitive, like it wasn’t hearing what it wanted to hear, and so it just kept kind of elaborating over and over.” (CG 26)  “They’re going back and asking the same questions, but they just turn them around.” (CG 03) |
| **Did you feel comfortable answering the AI’s questions?** | |
| General comfort and safety | “I felt comfortable. Yes.” (CG 03)  “Yes, I did. I feel really comfortable.” (CG 07)  “Yes, it made it seem they cared about my experiences.” (CG 09) |
| Comfort associated with familiarity and perceived relevance | “Yes, I felt comfortable, because questions that my daughter’s doctor asked me.” (CG 16)  “Yes, because the questions were relevant to my daughter.” (CG 23) |
| Comfort associated with the non-invasiveness of questions | “Yes, I feel comfortable as well answering all the questions. It wasn’t too invasive.” (CG 21)  “It wasn’t asking me anything that was uncomfortable or too invading.” (CG 26) |
| Comfort supported by human assistance and human-like interaction | “Because they took the time to try to help me. I was comfortable, yes.” (CG 08)  “It felt like I was talking to a person, an old school conversation with an older person.” (CG 12) |
| Increased comfort over time through a learning process | “At first, I did [feel uncomfortable], because AI. But then I started; it became better. I’m gonna give it a four.” (CG 22)  “Once I got the hang of it, it was pretty self-explanatory.” (CG 15) |
| **What did you like most about your experience?** | |
| Overall positive experience | “Everything I love it. Keep this as moving forward.” (CG 09)  “That it was a nice interview. I enjoyed it.” (CG 13)  “The tool works amazing. It’s good.” (CG 14) |
| Accessibility and voice features | “It read out to me what it was asking … pictures, stuff like that.” (CG 06)  “How it read to me, and it asked questions.” (CG 07) |
| Human-like conversation | “It made me feel I was talking to a person.” (CG 12)  “I felt like it was like it was a human. I would not have guessed that that was a computer.” (CG 12)  “Like the conversation, even though it was AI, it was still asking me the right questions.” (CG 19) |
| Clarity and organization of questions | “They were simple questions, easy to answer, easy to comprehend.” (CG 21)  “Everything was in order.” (CG 08) |
| Responsiveness | “I like the quick response, and I like how it understands. It does stay on topic.” (CG 22)  “I like how it responds to what you’re saying, like it understands actually, and asks you questions of what you say.” (CG 24)  “The fact that the computer … feedback was like, right on point.” (CG 14) |
| **What did you dislike or want improved?** | |
| Overall positive experience | “Nothing. I didn’t dislike anything.” (CG 01)  “No improvements. I didn't dislike anything.” (CG 05)  “No, I liked everything about the computer.” (CG 07) |
| Repetition or reiteration of questions | “Just the repetitious part. You can’t keep reiterating the question in different words.” (CG 12)  “Definitely just the elaborating. Once I answered, it’s best to move on.” (CG 26) |
| Technology challenges and limited computer literacy | “I’m not good with it. I call my grandkids for everything.” (CG 03)  “The only thing I disliked was the computer sometimes. If I tapped it too hard, it wouldn’t record my message.” (CG 15)  “Because if I’m actually with a different computer, I don’t know what to do.” (CG15) |
| Language difficulties | “I just don’t understand some of the words. I can’t read them all right.” (CG 08) |
| Errors and AI limitations | “The errors. maybe the AI needs to be updated.” (CG 04)  “It didn’t distinguish when we were talking about the housing authority.” (CG14)  “Typing session? Spelling was a little off, but I got it.” (CG 20) |
| Neutral responses | “No, I liked it. It was okay. I may so be neutral on it.” (CG 23)  “Nothing.” (CG 28) |
| **Which do you prefer—human or AI interviewer?** | |
| Preference for human interaction | “Because if I don’t understand anything, the human will tell me what it’s about.” (CG 03)  “I prefer human because I’m a people’s person.” (CG 04)  “Because I’d get more out of a person if I’m face to face.” (CG 07) |
| Preference for AI | “AI. It doesn’t judge you as a person would.” (CG 09)  “AI, definitely. It just straight up tells you the truth, doesn’t beat around the bush.” (CG 27) |
| Preference for both—complementary strengths | “Both. The computer wasn’t bad, but people understand feelings more.” (CG 06)  “Both … knowing that they [humans] did research makes me feel better.” (CG 11)  “It’s no different, but I just like to see a live person, too.” (CG 10)  “I don’t mind AI, obviously.” (CG 21) |
| AI acceptance and openness to future use | “This is actually my first good AI experience. I love a good human before this moment, but maybe you can give it a try now.” (CG 12)  “AI was giving key details … neutral on both ends.” (CG 23) |
